# Sustainable swine feeding programs require the convergence of multiple dimensions of circular agriculture and food systems with One Health

**DOI:** 10.1093/af/vfac077

**Published:** 2022-12-14

**Authors:** Gerald C Shurson, Pedro E Urriola

**Affiliations:** Department of Animal Science, University of Minnesota, St. Paul, MN, USA; Department of Animal Science, University of Minnesota, St. Paul, MN, USA

**Keywords:** circular agriculture, environmental impacts, multi-objective feed formulation, swine nutrition

**Implications**

- Sustainable pork production must evolve from a linear supply chain towards a circular system that integrates and optimizes multiple environmental, human and animal health, societal, and economic factors.
- Improving carbon, nitrogen, and phosphorus utilization efficiency of pork production requires new approaches for sourcing feed ingredients, formulating diets, precision feeding, and upcycling nutrients from food waste streams.
- One Health, circular, and sustainable swine feeding strategies can provide complementary or synergistic benefits for optimizing nutrition-health-environment interactions but widespread implementation is needed.
- Interdisciplinary and transdisciplinary research and education is needed to develop and implement meaningful sustainability action plans in the global agriculture and food industry.

## Introduction

Global agriculture and human food system is at a critical point for achieving future food security and sustainability because of destruction of soil, loss of biodiversity, degradation and pollution of water and land resources, and inefficient use, recovery, and recycling of nitrogen (N) and phosphorus (P) which have exceeded Earth’s planetary boundaries (UN/DESA, 2021). Many interrelated but separate global initiatives, such as the United Nations Sustainable Development Goals and International Nitrogen Initiative; circular economy, food system, and agriculture models; regenerative agriculture approaches; and One Health goals provide guidance for improving the four major components of sustainability—human, social, economic, and environment. All of these initiatives indicate that we need to modify pork production systems to become more sustainable, with circularity of nutrients and One Health goals as core components of these more sustainable systems. To create meaningful improvements in pork production sustainability, we need to think differently by progressing from using multidisciplinary (use of knowledge from different independent disciplines) approaches towards interdisciplinary (coordinated analysis and synthesis of knowledge between disciplines) and transdisciplinary (transcends traditional boundaries by integrating environmental, social, and health sciences) approaches for solving complex sustainability problems.

**Figure 1.**
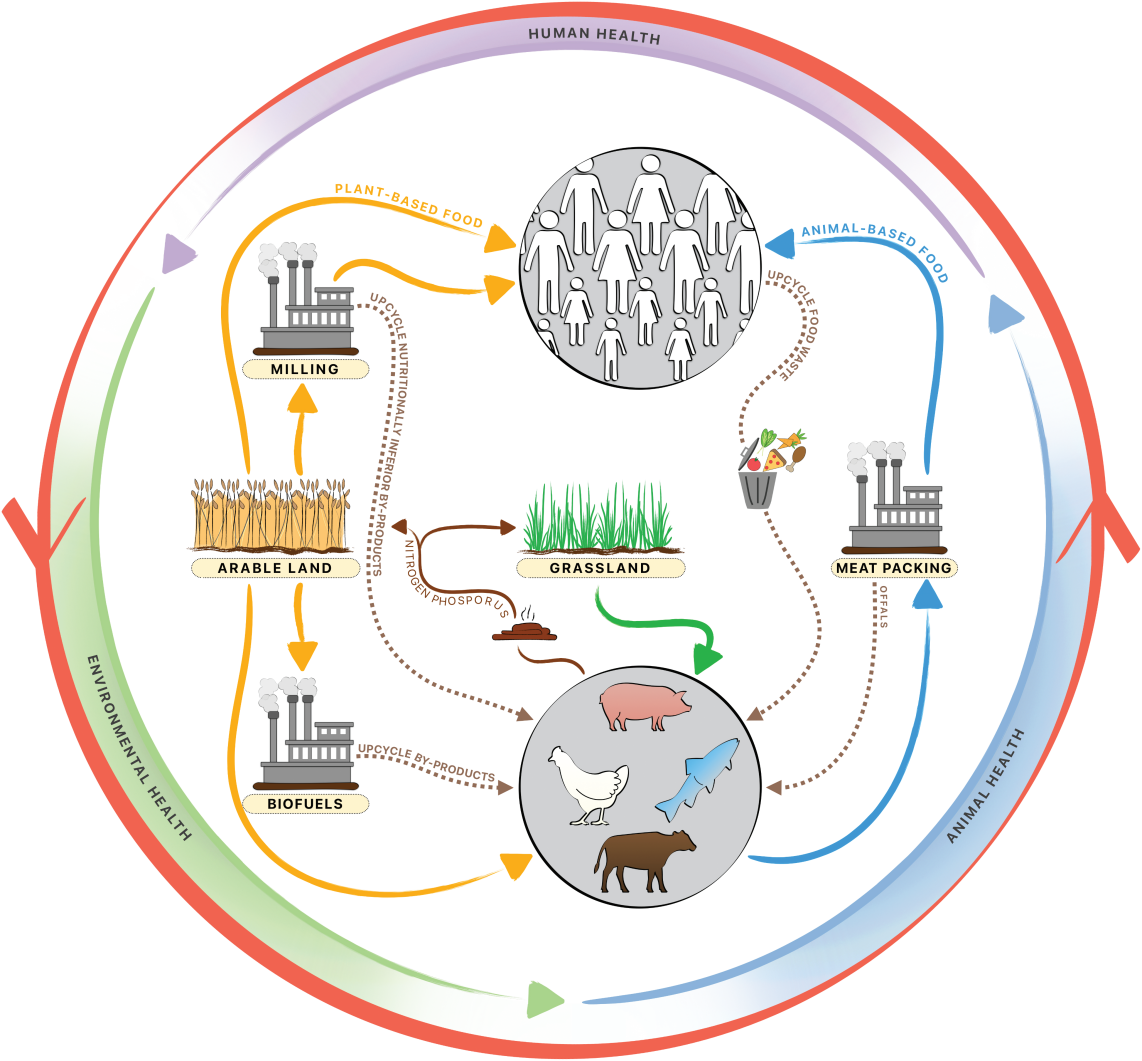
Sustainable pork production requires progressing from a linear supply chain toward a circular food system that integrates and optimizes environmental, animal, and human health.

The current food and agriculture supply chains are inefficient because they function as linear models comprised of independent actors attempting to achieve the greatest economic benefits in an extraction–production–consumption–discard manner at significant environmental costs (Figure 1). Therefore, circular agriculture and food supply chain models need to be developed to 1) implement technologies and practices that minimize the use of finite (e.g., land and water) and destructive resources (e.g., antibiotics, synthetic fertilizer), 2) encourage use of regenerative resources (e.g., animal manure, cover crops), and 3) close nutrient loops that prevent these valuable natural resources from leaving the food system and recycling nutrients back into production of more products. Although the global feed industry has a long history of recovering and recycling raw materials from agro-industrial and biofuels processes for many decades (Coffey et al., 2016), an increased focus on processes of origination and level of direct competition with food production has been initiated by FEFAC (2022) to optimize the recovery and recycling of nutrients from food and biofuels by-products into animal feed.

Pork production is the largest contributor to global meat production, and future consumer demand for pork is expected to increase (MacLeod et al., 2013). Although greenhouse gas (GHG) emissions from global pork supply chains (9.5%) are substantially less than those from dairy (30.1%) and beef (35.3%) supply chains, emissions will continue to increase unless mitigation strategies are implemented. The greatest opportunity for reducing the environmental footprint of pork production is to focus on swine feed composition and production because it is the largest contributor and accounts for 55% to 75% of the climate change impacts, 70% to 90% of energy use, and 85% to 100% of land use attributed to pork production systems (MacLeod et al., 2013; Dourmad et al., 2014; FAO, 2018). Therefore, to prevent further environmental degradation and inefficiencies, pork production systems must adopt a more circular agriculture model (Velasco-Muñoz et al., 2021; Marinova and Bogueva, 2022) that not only focuses on increasing productivity (i.e., producing more pork with fewer resources), but also minimizes amounts of external inputs, closes nutrient loops, and minimizes the environmental footprint.

One Health goals must also be achieved in sustainable pork production systems and have been used to evaluate and compare positive and negative impacts of future scenarios of improved pig production (Zira et al., 2022). One Health has been defined as “a collaborative, multisectoral, and transdisciplinary approach—working at the local, regional, national, and global levels—with the goal of achieving optimal health outcomes recognizing the interconnections between people, animals, plants, and their shared environment” (CDC, 2022). Several key issues of One Health include antimicrobial resistance, animal diseases, food safety, food security, and environmental contamination (CDC, 2022) directly apply to sustainable pork production systems. Unfortunately, large amounts of antimicrobials continue to be used in pig production systems around the world (Lekagul et al., 2019). Deficiencies in implementation and enforcement of current U.S. regulatory policies on antimicrobial use in meat and poultry production have limited the effectiveness of interventions compared with those in Denmark and the Netherlands (Wallinga et al., 2022). Because antimicrobial resistance continues to be a major health threat to humans, animals (pigs), the environment, and sustainability (Aarestrup et al., 2008), more effort is needed to reduce antimicrobial use in food animal production globally. Sources and types of feed ingredients, along with feed formulation strategies, are major contributors to nutritional efficiency, biosecurity and health, environmental footprint, and cost in pork production systems, we need to develop a more holistic approach for designing swine feeding programs that integrate One Health-circular agriculture and food systems goals and approaches.

## Ingredient Sourcing Matters

The introduction of a foreign animal disease, such as African Swine Fever virus (ASFV), to a major pork producing country (e.g., United States) can have numerous detrimental effects on sustainability including huge economic losses (Carriquiry et al., 2020), inability to export pork, loss of livelihoods, environmental pollution from carcass disposal caused by high mortality or euthanasia, disruption of feed and pork supply chains and rural communities. Worldwide, the ongoing spread of ASFV, and the potential for transmission through feed ingredients has led to increased biosecurity concerns, which involve recommendations for sourcing imported feed ingredients from non-ASFV infected countries and avoiding the use of porcine derived by-products and food waste in swine diets (Shurson et al., 2021a). However, there is no surveillance system to determine the prevalence of contamination, nor the concentration or infectivity of ASFV or other swine viruses in the global feed industry, which has led to high uncertainty of possible risk of transmission (Shurson et al., 2021a). Because of this high uncertainty, and the discovery that various swine viruses can survive for varying lengths of time in different feed ingredient matrices (Dee et al., 2018), biosecurity protocols have begun to be developed and implemented along entire feed ingredient supply chains.

Because of the perceived risk of ASFV and other foreign virus transmission through feed, demand for using rendered animal by-products and various sources of food waste in swine diets to reduce feed costs, improve nitrogen and phosphorus recovery, and reduce the environmental footprint has been diminished (Shurson, 2020). Furthermore, opportunities to capture nutritional efficiency, health, and environmental benefits of feeding spray dried animal plasma to weaned pigs (van Dijk et al., 2001) have not been fully realized because of perceived risks of transmission of swine viruses such as Porcine Epidemic Diarrhea virus (Shurson et al., 2021b).

Disruptions in “just-in-time” delivery of feed ingredients in global supply chains in recent years have led feed manufacturers to attempt to develop shorter supply chains that often include a desire to use more locally produced grains and oilseed meals compared with imported ingredients to provide a more reliable supply. Depending on the environmental impacts associated with using local feed ingredients, there may also be opportunities to feed diets with a lower environmental footprint to reduce overall environmental impacts of pig production (de Quelen et al., 2021), while also minimizing the risk of introducing foreign animal diseases. However, commodity traders play a key role in feed ingredient supply chain governance to ensure biosafe and deforestation-free sourcing of feed ingredients because indirect feed ingredient sourcing through intermediaries is a major problem for sustainable sourcing initiatives (zu Ermgassen et al., 2022).

**Figure 2.**
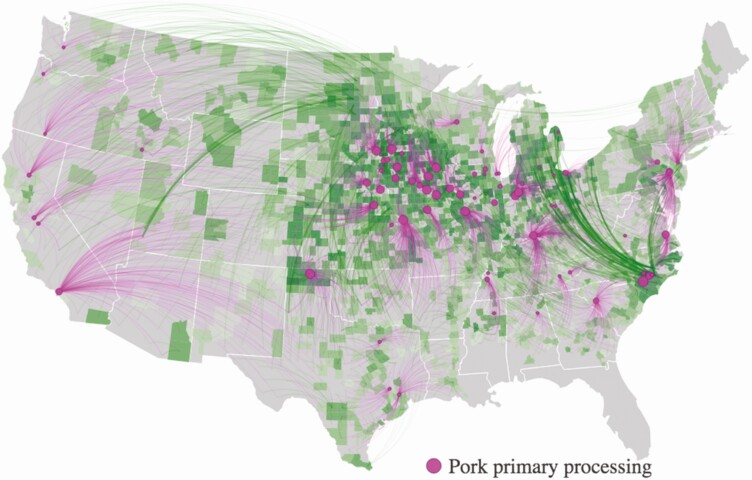
Pork supply chain connections linking corn production with downstream demand in 2012 where the magenta-colored arcs represent counties from which pigs are estimated to be sourced, and green arcs represent where embedded corn is estimated to be sourced used as feed in U.S. pork production systems. Dots represent locations and relative capacity of pork processing facilities. The darker shaded green regions indicate greater quantities and lighter green shaded regions indicate less quantities of corn sourced for feed in pork production systems (from Smith et al., 2017).

**Figure 3.**
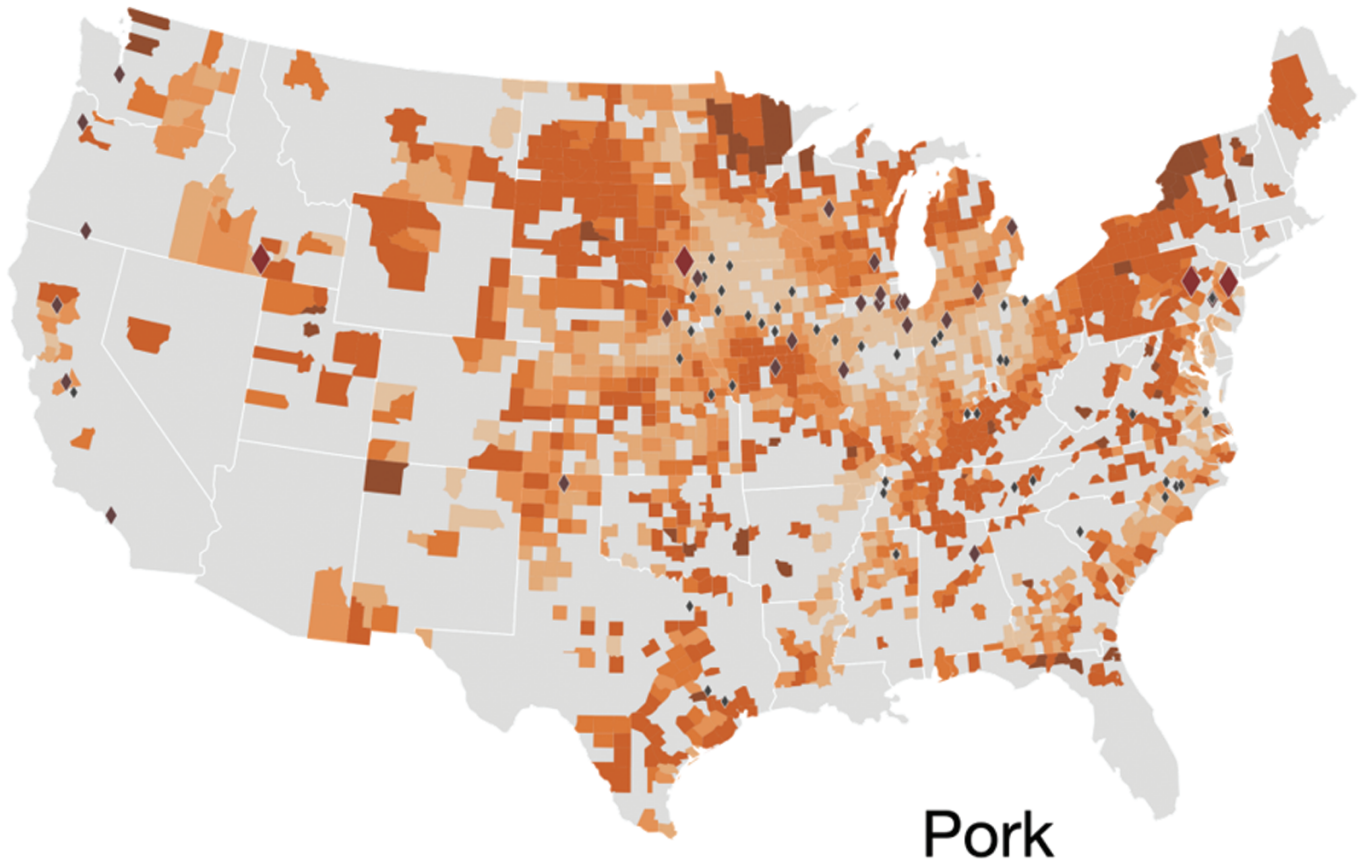
Land use change associated with corn production expansion and associated GHG emissions (CO_2_ equivalent) attributed per tonne of feed used in U.S. pork production systems. The darker shaded orange regions indicate greater quantities and lighter orange shaded regions indicate less quantities of corn sourced for feed in pork production systems. Diamonds represent pork processing facilities (from Pelton et al., 2021).

**Figure 4.**
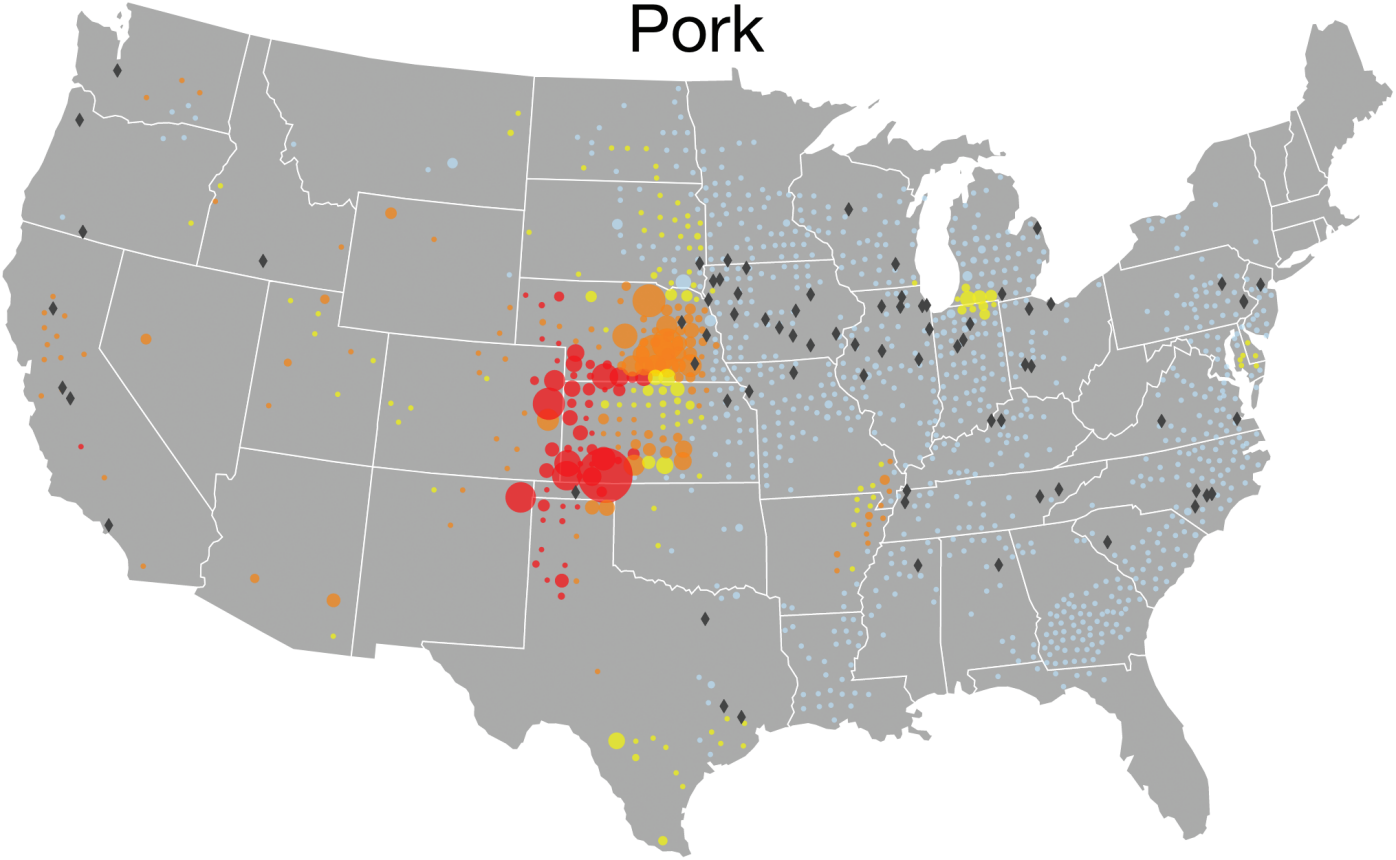
Embedded irrigation water consumption used to produce corn and soybeans for feed used in U.S. pork production systems and associated water depletion levels. Red circles indicate chronic, orange circles indicate seasonal, and yellow dots indicate dry-year water depletion levels (from Brauman et al., 2020).

**Figure 5.**
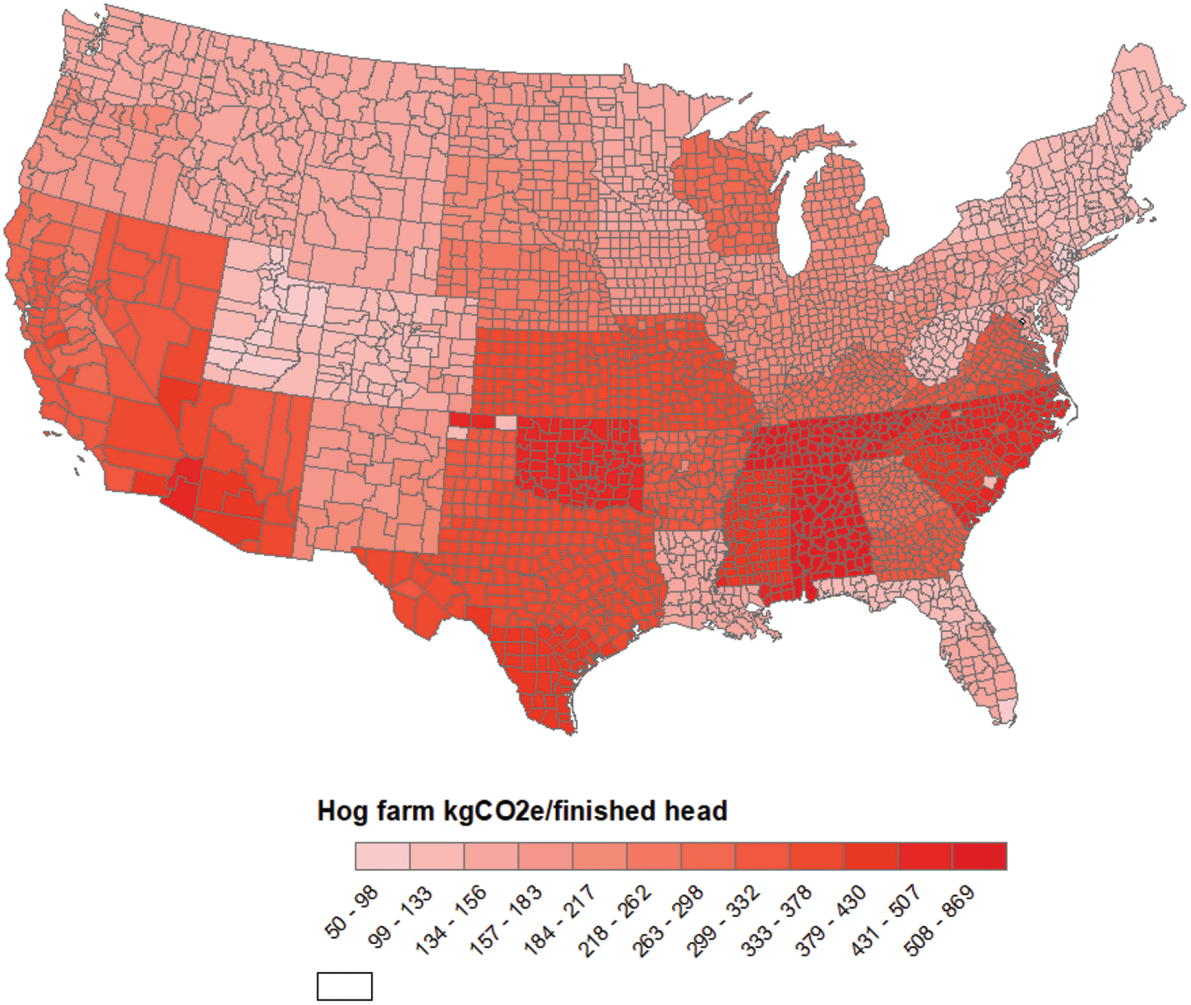
Comparison of greenhouse gas emission intensity (kg CO_2_ equivalent) for the production of market hogs among U.S. states.

The Global Feed LCA Institute (GFLI; https://tools.blonkconsultants.nl/tool/16/) has developed a large feed ingredient database consisting of 18 Life Cycle Assessment (LCA) environmental impact variables (Table 1) based on country of origin, and has the most widespread global application (European Union, USA, Canada). Although there are substantial differences in environmental impacts of feed ingredients at the national level, there are also substantial differences in environmental impacts of feed ingredients at the subnational level (i.e., state or province). For example, a FoodS^3^ model has been developed to quantify commodity flows and GHG, land use, and water consumption impacts at the county level within states in the United States to assist downstream actors in understanding upstream environmental impacts of major agricultural commodity supply chains (Smith et al., 2017; Pelton, 2018; Brauman et al., 2020; Pelton et al., 2021). As shown in Figure 2, corn production at the county level is linked with downstream demand for use in feed on swine farms at the county level, along with corn embedded in market hogs shipped to primary slaughter and processing facilities allow quantification of nutrient flows, GHG emissions, and land and water resources allocated to pork production at the subnational level (Smith et al., 2017). There are also substantial differences in land expansion and conversion to corn production used as feed in pork production systems and associated GHG emissions per tonne of feed (Figure 3), and some locations have chronic and drought year water depletion levels associated with embedded irrigation water consumption used to produce corn and soybeans for feed consumed in U.S. pork production systems (Figure 4). As a result, corn and soybean meal flows from production regions to consumption regions impact greenhouse gas emissions in pork production systems to varying degrees among states (Figure 5). Although it is possible to substantially reduce environmental impacts of swine feeding programs by sourcing major feed ingredients from countries or subnational locations of origin that have lower environmental footprints compared with other locations, feed ingredients with high environmental footprints (e.g., Brazilian soybean meal produced in the deforested region of the Amazon) will still need to be used for some productive purpose if circularity in global agriculture is going to be achieved.

**Table 1.** Summary of Global Feed LCA Institute environmental impact measures and their functional units that have been applied to feed ingredients

Environmental impact measure	Functional unit per kg product	Description
Global warming with or without land use change	kg CO_2_ equiv.	Indicator of potential global warming due to emissions of greenhouse gases to the air, using carbon dioxide as a standard, with or without a change in land use
Stratospheric ozone depletion	kg CFC11 equiv.	Indicator of emissions to air that cause destruction of the stratospheric ozone layer using chlorofluorocarbon-11 as a reference standard
Ionizing radiation	kBq Co-60 equiv.	Impact on radiation as measured by kilobecquerels of cobalt-60 radioactive isotope as a reference standard
Ozone formation, human health	kg Nox equiv.	Impact on nitrous oxide gases that affect the ozone and human health
Fine particulate matter formation	kg PM2.5 equiv.	Impact on air quality as atmospheric particulate matter with particles having a diameter of less than 2.5 micrometers
Ozone formation, terrestrial ecosystems	kg Nox equiv.	Impact on nitrous oxide gases that affect the ozone and human health
Terrestrial acidification	kg SO_2_ equiv.	Indicator of the potential acidification of soil and water due to the release of nitrogen oxide and sulfur oxide gases
Freshwater eutrophication	kg P equiv.	Indicator of the potential for increased phosphorus emission to freshwater
Marine eutrophication	kg N equiv.	Indicator of the potential for increased nitrogen emission to freshwater
Terrestrial ecotoxicity	kg 1,4-DCB	Impact of toxic substances emitted to the environment on land organisms using 1,4-dichlorobenzene as a standard
Freshwater ecotoxicity	kg 1,4-DCB	Impact of toxic substances emitted to the environment on freshwater organisms using 1,4-dichlorobenzene as a standard
Marine ecotoxicity	kg 1,4-DCB	Impact of toxic substances emitted to the environment on sea water organisms using 1,4-dichlorobenzene as a standard
Human carcinogenic toxicity	kg 1,4-DCB	Impact of carcinogenic toxic substances to the environment using 1,4-dichlorobenzene as a standard
Human noncarcinogenic toxicity	kg 1,4-DCB	Impact of noncarcinogenic toxic substances to the environment using 1,4-dichlorobenzene as a standard
Land use	m^2^a crop equiv.	Impact of converting nonagricultural land into agricultural use
Mineral resource scarcity	kg Cu equiv.	Indicator of depletion of natural inorganic mineral resources using copper as a standard
Fossil resource scarcity	kg oil equiv.	Indicator of the depletion of natural fossil fuel resources
Water consumption	m^3^	Indicator of the amount of water (cubic meters) required to produce a kg of product

## Nutritional Efficiency

Reducing GHG emissions associated with carbon utilization of feed ingredients can provide significant reductions in the environmental footprint of pork production, but there are additional challenges to environmental sustainability of pork production that extend beyond carbon emissions which include inefficient use of dietary N and P. Although methane and carbon dioxide are the major GHG emissions from ruminants, they are of less significance than nitrous oxide emissions in swine because of their greater effect on global warming potential. Nitrous oxide is present in the atmosphere at a lower concentration (6%) compared with methane (16%) and carbon dioxide (76%) but its global warming potential is nearly 10 times greater than methane and nearly 300 times greater than carbon dioxide (U.S. EPA https://www.epa.gov/ghgemissions/understanding-global-warming-potentials). The risk of abrupt environmental changes on Earth has increased because of human activity causing disruption of N and P flows from excessive waste (Li et al., 2019; Sutton et al., 2019). As a result, practices must be implemented to improve N (Uwizeye et al., 2020) and P (Oster et al., 2018) utilization efficiency, and reduce the C footprint of animal production systems (Gerber et al., 2014).

The global livestock industry contributes about one-third of human-induced N emissions (nitrates, ammonia, nitrous oxide, and other nitrogen oxides), with the poultry and pork supply chains contributing 29% of the total from food animals, and 68% of these N emissions are associated with feed production (Uwizeye et al., 2020). Nitrous oxide is a potent GHG, and ammonia and nitrogen oxides contribute to air pollution, cause acidification and eutrophication, and pose risks to human health (Galloway et al., 2008). Furthermore, nitrates and organic N have caused increased water pollution and biodiversity loss (Erisman et al., 2013). Globally only 20% of N is retained in useful products with 80% of various forms lost to the environment (Sutton et al., 2019). This inefficiency led the United Nation to launch a global initiative on Sustainable Nitrogen Management (UNEP, 2019), with efforts to mitigate nitrogen pollution mainly focused on enhancing the efficiency of N utilization in agricultural production (Liu et al., 2020).

Phosphorus is also a finite nutrient that has also exceeded planetary boundaries, caused by human activities that have disrupted biogeochemical flows (Steffen et al., 2015), and losses from agricultural activities (Rockström et al., 2009) contribute eutrophication of water systems resulting in algae blooms, oxygen depletion, death of fish, and creation of “dead zones” (Carpenter, 2008). Oster et al. (2018) identified several gaps that must be addressed to balance the agricultural phosphorus cycle to improve the sustainability of pig and poultry production and suggested improving animal feeding strategies (adding phytase to diets), reusing and recycling (manure and slaughter waste), focusing on soil agroecosystems, improving farmer economic performance, and developing effective government policies and regulations (P quota, P tax).

### Improving dietary nitrogen utilization efficiency

Several researchers have modeled dietary N utilization efficiency in swine by estimating the amount of N retained in the body, N excretion in feces and urine, and N emissions from manure as a percentage of total dietary N (Millet et al., 2018). Results from all of these studies show poor N efficiency (30% to 43%) as the amount of N retained as a percentage of total dietary N intake. Gerber et al. (2014) reported that N utilization efficiency for converting dietary N into edible food products in pigs (10% to 44%) was comparable to dairy cattle (15% to 35%), greater than in beef cattle (4% to 8%), but less than poultry (25% to 62%). Because 68% of total N emissions from food animal production is associated with animal feed, diet formulation strategies that minimize N waste offer the greatest opportunity for improvement in N utilization efficiency (Uwizeye et al., 2020).

Strategies to improve dietary N utilization efficiency include 1) the use of precision diet formulation to avoiding overfeeding protein above the pig’s requirements (Aarnink and Verstegen, 2007; Pomar and Remus, 2019), 2) precision feeding to match the nutrient requirements of individual pigs (Pomar et al., 2021), and 3) feeding low-protein diets supplemented with adequate amounts of crystalline amino acids (Wang et al., 2018; Pomar et al., 2021). Precision diet formulation and feeding is a key component of precision livestock farming with the goal of enhancing profitability, efficiency, and sustainability in the production of high quality and safe pork while achieving high animal welfare and minimizing impacts on the environment (Pomar and Remus, 2019). Precision feeding of pigs has been shown to reduce feed costs by more than 8%, N and P excretion by about 40% (Andretta et al., 2014), and greenhouse gas (GHG) emissions by 6% (Andretta et al., 2018).

Results from several studies have shown that dietary crude protein (CP) concentrations can be reduced by up to 4 percentage units when supplementing diets with L-lysine, DL-methionine, L-threonine, and L-tryptophan without compromising growth performance of pigs (Kerr, 2003). In general, for each one percentage unit decrease in dietary CP concentration, N excretion is decreased by about 10%, assuming that adequate crystalline amino acids are supplemented in the diet to meet the amino acid requirements of pigs (Kerr, 2003). Ammonia emissions from swine feces and urine can be reduced by 8% to 10% for each 10 g/kg reduction in dietary CP (Wang et al., 2018). Furthermore, minimizing excess dietary CP reduces the amount of undigested protein and amino acids available for fermentation by intestinal microbiota, which can reduce metabolites and the proliferation of *Bacteroides* and *Clostridium* species in the hindgut, thereby reducing the incidence of postweaning diarrhea and improving gut health in weaned pigs (Wang et al., 2018; Luise et al., 2021).

### Improving dietary phosphorus utilization efficiency

Like dietary N utilization, pigs also have low (34%) dietary P utilization efficiency (Gerber et al., 2014). To optimize P utilization when feeding diets containing plant-based ingredients with relatively high amounts of phytate to swine, exogenous phytase enzymes can be added to increase the proportion of dietary P used by the animal, reduce P excretion in manure, and minimize the antinutritional effects of phytate on digestibility of other nutrients (Shurson et al., 2021c). The addition of phytase to swine diets has been shown to increase P digestibility by 20% to 50% which subsequently reduces P excretion in manure (Lautrou et al., 2021). In addition, adding phytase to swine diets has been shown to reduce global warming potential by 17%, acidification potential (AP) by 110%, and eutrophication potential (EP) by 700% compared with unsupplemented diets (Nielsen and Wenzel, 2007). Although achieving “phytate-free” nutrition is possible, it will require strategic use of phytase in swine diets to account for the many interacting factors that limit its effectiveness (Cowieson et al., 2016). In contrast, rendered animal by-products contain no phytate, have relatively high concentrations of digestible phosphorus, and their use in swine diets should be increased to improve P recycling in a circular food system and because current rendering process conditions are adequate for destroying bacterial and viral pathogens of concern in pork production systems (Shurson et al., 2021b).

### Combined effects of feeding low crude protein diets supplemented with crystalline amino acids and phytase

An LCA study was conducted to determine environmental impacts of three swine diet formulation scenarios including standard base diets without or with supplemental crystalline amino acids, and the same base diets supplemented with synthetic amino acids and phytase for commercial pork production systems in Europe, North America, and South America of live pigs at the farm gate (Kebreab et al. (2016). EP was estimated to be reduced by 35% when feeding diets containing both crystalline amino acids and phytase, but the contribution from phytase was minimal (3%). This occurred because nitrogenous compounds dominated the contribution to EP compared with phosphorus, and it was assumed that soil P concentrations did not exceed the capacity for crop uptake, and the reduction of P in manure would be compensated using inorganic fertilizer. Kebreab et al. (2016) also reported that feeding the amino acid supplemented diets with or without phytase also provided significant benefits for reducing AP. Therefore, there are significant synergistic nutritional efficiency, health, and environmental effects of feeding low CP swine diets supplemented with adequate amounts of crystalline amino acids and phytase.

### The copper and zinc dilemma

The ban on using antibiotics as growth promoters in swine diets has led to the use of a plethora of alternative growth promoting feed additives, including pharmacological dietary levels of supplemental Cu and Zn, which have become preferred growth promoters because they provide relatively consistent and cost-effective growth promotion of weaned pigs compared with other types of feed additives (Dębski, 2016). Pharmacological dietary levels of Cu and Zn are considered functional nutrients because they have antimicrobial effects when added to diets in excess of their requirements (Shurson et al., 2021c). Feeding pharmacological doses of zinc (1,000 to 3,000 ppm Zn) has been an effective method of controlling postweaning diarrhea in pigs (Bonetti et al., 2021). However, when the high dietary levels of Cu and Zn are fed to newly weaned pigs, nearly all of the amounts consumed are excreted manure (Jondreville et al., 2003). As a result, natural decomposition of organic matter in liquid slurry is reduced, Zn and Cu accumulate in top soil from long-term manure application because manure application rates greatly exceed Cu and Zn uptake by crops, and Cu and Zn toxicity of plants and soil micro-organisms can occur (Jondreville et al., 2003). In addition, run-off to surface water and ground water contamination can occur (Dębski, 2016), and long-term use of high dietary levels of Cu and Zn may promote the spread of antimicrobial resistance in gut microflora of pigs (Holzel et al., 2012). Because of these environmental and health concerns, the European Union has banned the use of pharmacological doses of zinc oxide in pig diets beginning in June 2022 (Bonetti et al., 2021). Strategies to capture the benefits of using high dietary ZnO levels to control postweaning diarrhea in weaned pigs while minimizing the negative environmental impacts and contributions to antimicrobial resistance are needed to improve the sustainability of pig production.

## Multi-objective Feed Formulation

Improving the environmental sustainability of pork supply chains requires measurement of environmental impacts, establishing goals and time-based action plans to achieve them, and requires collaboration along all segments of the food chain from production to consumption (Smith, 2008). Many U.S. food companies have established sustainability goals and programs that require producers of food products to provide evidence of low environmental footprints, which have led to differentiating and preferentially selecting suppliers that conform to desired environment standards (Bezares et al., 2021). Because the origin of feed ingredient sources used, and the nutritional composition of swine diets have a significant effect on overall environmental impacts of pork supply chains, multi-objective feed formulation is an emerging approach being implemented in the global feed and pork industries. This approach uses LCA environmental impact data for feed ingredients as additional constraints when formulating least-cost, nutritionally adequate, low environmental impact diets (de Quelen et al., 2021). Considerations should also be given to adding diet formulation constraints on inclusion rates of feed ingredients that contain significant antinutritional factors that are detrimental to swine health (e.g., mycotoxins) and the environment (e.g., pharmacological levels of Cu and Zn), while promoting the use of functional ingredients (e.g., spray dried animal plasma) and nutrients (e.g., soy isoflavones) that enhance swine health and reduce the environmental footprint (Shurson et al., 2021c). Preferential use of biosecure feed ingredients that contain a high proportion of digestible N and P relative to total concentrations of these nutrients should also be part of multi-objective feed formulation to improve nutritional efficiency and circularity. In addition, using measurements that more accurately assess the nutri-physiological characteristics of feed ingredients to better predict and optimize physiological responses can further enhance nutritional efficiency beyond the use of traditional nutritional evaluation measures (Shurson et al., 2021c). For example, measures that indicate the physiochemical and fermentability effects of various types of dietary fiber; functional nutrients and ingredients; digestion kinetics of starch, protein, and lipids of feed ingredients; and circadian rhythm effects on feeding behavior and gut microbiome can further enhance nutritional efficiency in precision swine feed formulation and feeding programs (Shurson et al., 2021c).

## Increase Recycling and Upcycling Nutrients from Waste Streams

Multi-objective swine feed formulation that includes environmental impact constraints cannot completely achieve desired reductions in carbon, N, and P waste and emissions without creating new and improved sources of nutrients from circular agriculture, biofuels, and food systems. Historically, the feed industry originated in response to the need to redirect, recover, and “upcycle” valuable nutrients from by-products produced by the grain milling, meat packing, and milk processing industries into animal feeds to comply with laws prohibiting these waste streams from being discarded into water sources and causing environmental pollution (Coffey et al., 2016). Although raw materials from various waste streams in agriculture, food, and biofuels continue to be used in animal feeds to provide some circularity of nutrient use, more efforts are needed to further increase circularity of agriculture and food production (FEFAC, 2022) and to meet future demand for feed protein, which is becoming a deficit due to expansion of global animal production (Kim et al, 2019). Extensive research has been conducted to characterize the nutritional value, identify the benefits and limitations, and develop metabolizable energy and digestible amino acid prediction equations for corn distillers dried grains with solubles, a coproduct of U.S. ethanol production, can be used in precision swine diet formulations to increase nutritional efficiency (Urriola et al., 2014; Zeng et al., 2017; Jang et al., 2021). Similar investments in research are needed to develop methods for dynamically determining net energy and digestible amino acid and phosphorus concentrations in other major by-products used in swine diet to increase C, N, and P efficiency.

Furthermore, there is considerable interest in developing process technologies that upcycle nutrients from lower value discarded by-products into feed ingredients that have higher nutritional quality and value as part of a circular system for improved sustainability. Because 40% of food produced (more than 2.5 billion tonnes) is lost or wasted and not consumed globally (WWF, 2021), upcycling numerous pre- and post-consumer food waste streams into animal feed represent a significant opportunity to reduce N and P losses and GHG emissions (by diverting away from land fill disposal), as well as reduce land, water, and fossil fuels resource use attributed to animal feed (Shurson, 2020), if adequate thermal processing is used to destroy bacterial and viral pathogens before feeding to swine (Shurson et al., 2021b). Unfortunately, because of historical experiences of disease outbreaks linked to feeding uncooked “garbage” to pigs, the adoption of this high impact, environmentally sustainable feeding strategy has been constrained in the United States due to current federal and state regulations, lack of education and acceptance, and lack of industry infrastructure encourage entrepreneurship (Shurson, 2020; Dou et al., 2021).

New biotic processing methods (e.g., insects, microalgae, fungal fermentation) are emerging to upcycle nutrients from waste streams in animal production, food processing, and postconsumer food waste into higher value nutrient sources as animal feeds. For example, low value agri-food waste streams such as animal manure and postconsumer food waste are being used as substrates to produce insects which are more concentrated and highly palatable protein and energy sources for use in animal feeds (Makkar et al., 2014; Sogari et al., 2019). Selected microalgae strains can efficiently and economically use nutrients in dairy wastewater (Lu et al., 2016) and meat processing (Lu et al., 2015) streams to remove nitrogen and increase biomass yield, nitrogen, and oil concentrations for use in biofuels production and animal feeds. Furthermore, upcycling high fiber ingredients such as wet corn distillers grains with solubles, soybean hulls, cottonseed meal, and canola meal using solid state fungal fermentation can be an effective approach to degrade fiber, mycotoxins, and phytate while enhancing amino acid concentrations, balance, and digestibility (Barnhart et al., 2021; Sun et al. 2021a, 2021b, 2021c). Therefore, further development and scaling up ofthese biotic processing technologies can greatly enhance the nutritional value of relatively low value feedstuffs in swine diets and provide significant contributions towards reducing the environmental footprint of feed used in commercial pork production systems.

## Conclusions

Sustainable pork production requires increased attention to sourcing of feed ingredients, use of LCA and antinutritional factor constraints for feed ingredients in multi-objective feed formulation and precision nutrition practices, and further development and implementation of strategies to upcycle nutrients from nutritionally inferior by-products and food waste streams into swine feed ingredients to improve carbon, nitrogen, and phosphorus utilization efficiency and reduce the environmental footprint of pork production. Many of these strategies are complementary to achieving One Health and circular agriculture and food system goals but continued use of pharmacological dietary levels of Cu and Zn in weaned pig diets are not. Future swine feeding programs must be designed to not only be economical and optimize swine health and productivity, but they must also minimize risk of transmission of pathogens, avoid contributing to antimicrobial resistance, and reduce the environmental footprint of pork production.

*Conflict of interest statement.* None declared.

## About the Authors

**Shurson biosketch**

Dr. Gerald (Jerry) Shurson is a Professor in the Department of Animal Science at the University of Minnesota. He received his B.S. degree in Animal Science and Agricultural Economics at the University of Minnesota, and his M.S. and Ph.D. degrees in swine nutrition at Michigan State University. Jerry has a very diverse research program which involves determining biological mechanisms of nutritional responses in pigs; assessing the nutritional value of biofuels coproducts; feed safety, biosecurity, and environmental impacts of ingredients and supply chains; and swine nutrition-health interactions. He provides programmatic leadership for several interdisciplinary research teams at the University of Minnesota including the Integrated Animal Systems Biology team, African Swine Fever Response Team, and Animal Agriculture Sustainability team. His research program has generated $19 million in research funding and resulted in 175 scientific publications and numerous technical presentations to scientific, industry, and government audiences in 32 countries during his 36-year academic career.

**Urriola biosketch**

Dr. Pedro E. Urriola is Research Associate Professor at the Department of Animal Sciences of the University of Minnesota. Original from Venezuela, he studied the equivalent to Veterinary Medicine at the Universidad Central de Venezuela. He completed an MSc at the University of Minnesota, and a PhD at the University of Illinois. He worked as a Research and Development Manager at Cargill Animal Nutrition. Now, Pedro focuses on collaboration with experts in systems-biology, ecology, animal health, and food safety to develop solutions for sustainable food systems.
